# Health Inequalities amongst Refugees and Migrant Workers in the Midst of the COVID-19 Pandemic: a Report of Two Cases

**DOI:** 10.1007/s41649-021-00198-8

**Published:** 2022-01-15

**Authors:** Shu Hui Ng

**Affiliations:** Medical Law and Ethics (LLM), School of Law, University of Edinburgh, Edinburgh, UK

**Keywords:** Refugees, Migrant health, Inclusion, Healthcare access, Right to health, COVID-19

## Abstract

Malaysia hosts a significant number of refugees, asylum-seekers and migrant workers. Healthcare access for these individuals has always proved a challenge: language barriers, financial constraints and mobility restrictions are some of the frequently cited hurdles. The COVID-19 pandemic has exacerbated these existing inequalities, with migrants and refugees bearing the brunt of chronic systemic injustices. Providing equitable healthcare access for all, regardless of their citizenship and social status remains an ethical challenge for healthcare providers, particularly within the framework of a resource-limited healthcare system. Inclusive healthcare and socio-economic policies are necessary to ensure every individual’s equal opportunity to attain good health. The collective experiences of refugees and migrants in the pursuit of healthcare, as highlighted by the two cases described, showcases the importance of equity in healthcare access and the detrimental implications of non-inclusive healthcare and socio-economic policies.

## Introduction

In Malaysia, there are some 179,450 refugees and asylum-seekers registered with the United Nations High Commissioner for Refugees (UNHCR), of which 154,860 are from Myanmar, and the remaining 24,590 are individuals from 50 other countries fleeing war and persecution (UNHCR Malaysia, n.d.b). Malaysia is considered a major destination country for migrant workers, with official estimates quoting the number of foreign workers in Malaysia to be as high as 3 million, but the actual total number of foreign workers is postulated to be around 5.5 million (World Bank Group 2020, 17–18). The 1951 Refugee Convention states that refugees should enjoy access to health services equivalent to that of the host population (UNHCR Malaysia, n.d.c). However, Malaysia is not one of the signatories to the 1951 Convention Relating to the Status of Refugees and its associated 1967 protocol, making efforts to address the economic, healthcare and social needs complicated by numerous challenges (Chuah et al. 2018).

To begin with, there are distinct differences between refugees and undocumented migrant workers. Refugees are people who have fled war, violence, conflict or persecution to find security in another country, and cannot return home safely (UNHCR Malaysia, n.d.a). On the other hand, undocumented migrants are individuals who lack the proper authorization to enter or stay in the country. This includes migrants who were falsely promised work, victims of human trafficking or simply persons who have overstayed their visa (UNHCR Malaysia 2021). The Malaysian law does not clearly differentiate between refugees, asylum-seekers and undocumented workers, and all such individuals are broadly classified as and deemed ‘illegal immigrants’ (Yasmin et al. 2019). Refugees in Malaysia can and are still vulnerable to arrest for immigration offences (UNHCR 2011, 212). For the purpose of this article, they will be collectively described as ‘the vulnerable population’.

At present, there are limited healthcare financing options for refugees, asylum-seekers and migrant workers in Malaysia. In 2017, the UNHCR in collaboration with RHB Bank launched the Refugee Medical Insurance (REMEDI) for refugees, but only 12.2% of UNHCR cardholders were enrolled (UNHCR Malaysia, n.d.c). The low rate of enrolment and a high rate of payouts have incurred significant losses to the partnering bank, leading to the cessation of the insurance policy scheme from 16 June 2018 (Asia Pacific Refugee Rights Network 2018). To date, refugees registered with the UNHCR are accorded a 50% discount off the foreigner’s rate at Malaysian government healthcare facilities (Amara and Aljunid 2014). On the other hand, the Hospitalisation and Surgical Scheme for Foreign Workers (SPIKPA) was introduced as a mandatory healthcare financing system for all documented migrant workers. With an annual premium of RM 120 per migrant worker, the insurance scheme offers an annual coverage of RM 20,000. However, with increased non-citizens fees at public hospitals, the SPIKPA coverage is likely inadequate in providing financial risk protection (Loganathan et al. 2020).

Before the COVID-19 pandemic, the vulnerable population were already living in suboptimal living conditions with issues of overcrowding, poor sanitation and limited access to affordable healthcare (Spiegel et al. 2010). While refugees and migrant workers are able to seek medical attention in public or private healthcare facilities, equitable access to such services are often hindered by a variety of factors including the cost of treatment, language barriers and the restriction of mobility in the public due to a fear of discrimination or persecution (UNHCR Malaysia, n.d.c). The lack of inclusive policies which seek to ameliorate healthcare access to refugees and migrant workers has resulted in members of this diverse community being disproportionately affected by the COVID-19 pandemic. On 12 August 2021, a national record high of 93 ‘brought-in-dead’ (BID) cases were recorded, and 36 of them were foreigners (Kaos 2021). The following two case discussions depict the realities faced by the vulnerable population in Malaysia.

## Case 1: a Refugee with a Chronic Disease

Madam Syahirah is a 54-year-old refugee who has resided in Malaysia for more than two decades. She was diagnosed with type 2 diabetes mellitus and hypertension over 20 years ago and has been on oral medications. As a UNHCR-registered refugee, she is still required to pay for medical consultation and treatment whenever she goes for an appointment in a clinic. As refugees in Malaysia are denied the rights to seek legal employment, she has resorted to working odd jobs for decades, and this has left her without a stable source of income. Her limited financial situation has several knock-down effects, such as hindering her access to healthier dietary options, and making it impossible for her to afford the insulin that would help her achieve optimum disease control. Since the pandemic began last year, her financial circumstances have worsened as her family members and herself can no longer work or travel freely. They have become dependent on donations, and her dietary choices are limited to whatever little her family can afford. Her diabetic control went from being suboptimal to completely uncontrolled, and her disease has inevitably progressed. She now suffers from significant nephropathy, peripheral neuropathy and diabetic retinopathy—all significant and potentially avoidable complications of diabetes mellitus.

## Case 2: a Migrant Worker with Acute COVID-19 Infection

Mister Joseph is a 51-year-old migrant worker who has worked as a construction worker in Malaysia for the last two decades. He was made redundant from his job last year at the start of the pandemic and has faced immense challenges in finding new employment. In July 2021, he developed symptoms of shortness of breath associated with intermittent fever, both of which lasted a week. He avoided seeking medical attention promptly due to financial concerns, and his fear of persecution due to his legally obtained work permit being long expired. After a week of his illness, he was so breathless that he was unable to speak. His oxygen saturation levels were dangerously low, and a point-of-care COVID-19 antigen test done at a private general practitioner’s clinic yielded a positive result. An ambulance was called for immediately, but after more than an hour’s wait, he was informed that due to overwhelming demand the ambulance service was unable to allocate an ambulance for Joseph despite his critical condition. He was advised to either pay for a private ambulance service, or to present himself to the hospital via ride-hailing service with personal protective equipment (PPE). As Joseph was not able to pay for a private ambulance, he finally decided to present himself to the hospital via ride-hailing service after much persuasion. Fortunately, after days of oxygen supplementation, his condition gradually improved and he was discharged home.

The above two cases were composite experiences of multiple patients I have encountered in my clinical practice. Patient’s names and demographics have been changed, and their respective disease clinical courses have also been altered to eliminate any possible identifying features. The above cases serve to illustrate the difficulties faced by the refugee and migrant population in seeking both acute and long-term healthcare, and how this has been aggravated by the pandemic, amplifying the impact of non-inclusive legal, health and social protection policies on their health.

## The Right to Health

The WHO Constitution (1946) envisioned ‘…the highest attainable standard of health as a fundamental right of every human being’, and the ‘right to health must be enjoyed without discrimination on the grounds of race, age, ethnicity or any other status’. (WHO 2017). Malaysia has a robust health system with universal health coverage, in which the public healthcare service is funded by general taxation and health services are heavily subsidized for its citizens and residents (Chua and Cheah 2012). Seventy-five percent of the country’s hospitals are within the highly subsidized public sector, whereby Malaysian citizens pay as little as RM 1 (USD 0.30) for a general outpatient consultation and RM 5 (USD 1.50) for specialist-level medical care (Jaafar et al. 2013, 44). However, such comprehensive healthcare coverage does not extend to the vulnerable population of refugees and undocumented immigrants—a group who have been denied the legal rights to seek employment for independent financial sustenance. Despite the 50% discounted foreigner rates extended by the Malaysian government to refugees in need of treatment at public health facilities, the fees still remain prohibitively high for members of this vulnerable population (Médecins Sans Frontières 2019b). Furthermore, there is a huge disparity in the healthcare charges for citizens and non-citizens (Hospital Kuala Lumpur 2020), making healthcare fees exorbitant for refugees and undocumented migrants. As legal documents are required on presentation to healthcare facilities, undocumented migrant workers and refugees often feel stigmatized and discriminated against and are especially fearful of arrest, detention and deportation (Loganathan et al. 2019). In addition, healthcare professionals from the public healthcare facilities are informed via a circular issued by the Ministry of Health that the presence of undocumented migrants who seek treatment in said healthcare facilities will need to be reported to the immigration authorities (Ministry of Health Malaysia 2001). Such policies, when implemented and enforced, create a climate of mistrust and apprehension amongst undocumented migrants and refugees, which serves to further restrict their access to health care.

Without the legal right to work and equitable entitlement to public welfare services, refugees and undocumented migrants often engage in informal employment (Todd 2019). In a survey done by the Malaysian Human Rights Commission, 26% of refugees earned less than RM 500 per month, 58% earned between RM 500–1000 per month, whilst the remaining had no fixed income (SUHAKAM 2013, 25). The implementation of nationally enforced lock-down measures during the pandemic has resulted in most of them who took on unregulated job for a daily wage become unemployed and thus highly reliant on financial handouts from non-governmental organizations and charitable members of the community. In the absence of basic financial security to sustain access to food and housing, healthcare is often prioritized last.

Even prior to the pandemic, the access to healthcare refugees and undocumented migrants were hindered by various socio-economic determinants and a non-inclusive legal environment, compounded by other factors such as poor health literacy, communication impediments and cultural differences (Chuah et al. 2018). As a result of the pandemic, this vulnerable population faced even more overt challenges. When faced with chronic diseases which require long-term follow-up and treatment compliance, families are forced to make difficult choices between healthcare, food and housing. As illustrated by the case of Madam Syahirah, inadequate lifestyle modification, coupled with inconsistent access to follow-up care and medications, has contributed to the progression of her chronic illnesses and led to undesired long-term disease sequelae. Such complications are potentially irreversible and were even preventable should she have access to consistently available and quality healthcare. In reality, the management of these disease-related complications would actually incur more financial burden and thus further disincentivizes good health-seeking behaviour.

The implementation of health justice requires us to address the exclusion from social opportunities and healthcare access in an inclusive manner. One could argue that because refugees and undocumented migrants do not pay taxes, would it then be morally and ethically justifiable for them to receive equivalent healthcare as citizens? It is imperative to realize that marginalization of certain populations on the basis of migration status serves to expose vulnerable people to higher rates of illness, which is largely associated with lifestyle and behavioural factors, as well as access to affordable healthcare. If policies are not structured to provide equivalent health entitlement for all, then perhaps the socio-economic construct should aim to empower members of these vulnerable populations to have the opportunity to seek legal employment and, thus, generate the financial capacity to provide for their own health care needs.

## The Need for Inclusive Policies

The COVID-19 pandemic presented most healthcare systems with myriad unprecedented challenges—straining healthcare resources and exacerbating the existing obstacles refugees and undocumented migrants face in securing healthcare access. This is exemplified by hampered efforts to include refugees and undocumented migrants in the national vaccine roll-out scheme which were the product of heightened immigration arrests and anti-foreigner rhetoric (Fishbein and Hkawng 2021). The fear of persecution has kept many undocumented migrants from accessing health care or registering themselves for the national COVID-19 vaccination drive. Mixed messages from the Malaysian government regarding refugees’ and migrants’ access to COVID-19 vaccinations—certain quarters have encouraged refugees and migrants to step forward and get vaccinated, while others have threatened detention should they do so—have contributed towards an atmosphere of uncertainty amongst this vulnerable population. This distrust is further exacerbated by recently conducted immigration raids by the Malaysian authorities which rounded up hundreds of individuals, including those with UNHCR identification (Fishbein and Hkawng 2021).

For these members of the vulnerable population, the trepidation of falling sick in a foreign land is compounded by the inevitable financial burden associated with seeking medical care, and the threat of immigration-related reprisals. A pervasive sense of insecurity has resulted in their reluctance to seek medical attention, even in emergencies (Médecins Sans Frontières 2019a). A local study which sought to explore barriers to healthcare faced by documented and undocumented migrant workers revealed that ‘undocumented migrants are likely to avoid necessary hospital care and are often only brought in to hospital either unconscious or critically ill’ (Loganathan et al. 2019). As illustrated in the case of Joseph, fear was an important factor deterring him from seeking early medical attention, to the point of significant clinical deterioration. His worries over arrest or detention far exceeded concern for his own health and well-being. In a highly infectious pandemic, the fear of immigration reprisals will not address human security and healthcare concerns of refugees and migrants (Daniel and Jeffrey 2020), but instead would negatively impact the ability to conduct contact tracing and contain the spread of infectious diseases.

## Conclusion

The allocation of medical resources and immigration policy have always been contentious issues in Malaysia, particularly in circumstances where their spheres of influence intersect. Decades of efforts by dedicated individuals and various advocacy groups have yet to evolve into more inclusive healthcare policy making which seeks to improve the difficulties faced by the vulnerable population. Migrant workers and refugees are members of our community, and thus, the barriers to healthcare should be seen as a reality to be managed with comprehensive healthcare policies that form the backbone of a more sustainable and equitable healthcare system. It is crucial that refugees, asylum-seekers and the migrant population receive equal entitlement and access to healthcare. Without tangible efforts from all stakeholders to develop a nuanced, pragmatic and coherent policy response to the growing need for non-discriminatory healthcare access, migrants and refugees will continue to struggle for their quest to equitable health care.
